# Clustering Cortical Rhythms: Monoaminergic Signatures in Time-Frequency EEG Dynamics

**DOI:** 10.3390/biomedicines13081973

**Published:** 2025-08-14

**Authors:** Vasily Vorobyov, Alexander Deev

**Affiliations:** 1 Institute of Cell Biophysics, Russian Academy of Sciences, 142290 Pushchino, Russia; 2 Institute of Theoretical and Experimental Biophysics, Russian Academy of Sciences, 142290 Pushchino, Russia; aadeev@gmail.com

**Keywords:** electroencephalogram, EEG, frequency, clustering, agonist, antagonist, quipazine, cyproheptadine, SKF-38393, SCH-23390, clonidine, yohimbine

## Abstract

**Background**: Multiple studies of the role of neurotransmitter systems in the effects of various substances on brain functions under normal conditions and at various brain disorders have demonstrated the relatively high usefulness of the electroencephalogram (EEG). However, little is known about EEG “fingerprints” of direct neurotransmitter–receptor interactions, in particular, for monoamine (MA) systems involved in the main brain functions. **Methods**: We looked at how the EEG effects of serotonin, dopamine, and norepinephrine receptors activating substances (quipazine, SKF-38393, and clonidine, respectively) injected into the brain’s lateral ventricles were affected by corresponding blockers (cyproheptadine, SCH-23390, and yohimbine) in freely moving rats. We introduced a method for clustering significant changes in the EEG spectra based on specific time intervals and narrow frequency subranges. **Results**: Stimulating serotonin and dopamine receptors caused specific suppression of EEG activity around 10 Hz and an increase near 18 Hz, respectively. The effects were reduced after pretreatment with the corresponding receptor blockers. Clonidine produced clusters of increased and decreased EEG activity around 6 Hz and 21 Hz, respectively, which were weakened by the blocker, yohimbine. These results demonstrate the “signatures” of different MA systems in EEG time–frequency clustering. **Conclusions**: We consider the developed approach as a potentially useful tool in clinics for evaluation of MA transmission pathology and its therapy with corresponding substances penetrating the blood–brain barrier.

## 1. Introduction

The search for reliable indicators of neurotransmitter system activity in the brain is essential for understanding the broader neurochemical mechanisms underlying its function. This need is becoming increasingly urgent due to the widespread and growing use of pharmacological drugs, the rapid emergence of new bioactive compounds, and ongoing environmental pollution, including electromagnetic radiation, which in turn affects the pharmacological intrusions into the brain. As the functioning of the brain is based on a coordinated activity of its neurochemical systems, any changes in their functional states are inevitably accompanied by modification of the brain’s reactions to external stimuli. While previous studies have shown the involvement of neurotransmitter systems in the brain’s response to these factors, most of the evidence has come from biochemical and behavioural research. However, these approaches present certain challenges: biochemical studies often involve complex, long-term recordings that are difficult to interpret, while behavioural studies reflect highly integrated brain activity, making it hard to isolate specific neurochemical mechanisms.

An alternative and widely used method is the recording of brain electrical activity through electroencephalography (EEG), which does not share many of these limitations. Among EEG features, its frequency composition—or spectrum—has proven particularly useful in evaluating the effects of pharmacological compounds and neurotoxicants on brain function [1,2,3,4,5,6], as well as their impact on neurotransmitter systems [7,8,9,10,11,12,13]. From a methodological standpoint, the key role of neuronal membrane potentials in generating EEG activity [14] and their close relationship with neuromodulators in the brain [15] support the use of EEG in this area of research.

Disruptions in neurotransmitter systems—particularly the monoaminergic (MA) systems—have been linked to emotional dysregulation, depressive disorders [16], and stress-related conditions [17]. Additionally, classical psychedelics used in treating psychiatric disorders are known to affect MA neuronal activity [18]. MA systems are also considered to play a central role in the early stages of Alzheimer’s disease [19] and cognitive impairments in Parkinson’s disease, which are often accompanied by EEG slowing [20]. In patients with major depressive disorder, characteristic frontal alpha asymmetry in EEG recordings has been shown to be modulated by the serotonergic and noradrenergic systems [21]. Moreover, based on the idea that impaired error monitoring is a core feature in various psychiatric conditions, Barnes and colleagues [22] showed that this deficit could be improved by enhancing catecholamine function. Overall, the relationship between EEG frequency spectra and monoaminergic system activity could serve as a valuable marker for neuromodulator balance, particularly in conditions involving memory impairments and/or mental illness [23].

However, research in this field varies widely in methodological design—including differences in animal models, used substances, dosages, administration routes, observation times, and control conditions. These discrepancies may explain conflicting findings across studies and make it difficult to apply their results in broader analyses of neurochemical mechanisms, whether in disease or in response to external exposures. Furthermore, many studies do not present complete protocols for evaluating dose–response relationships, nor do they consistently analyze the separate and combined effects of agonists and antagonists. This is essential to confirm that observed EEG effects are specifically mediated by neurotransmitter–receptor interactions. A major gap in the field is the lack of direct comparative studies on how different neurotransmitter systems contribute to EEG frequency composition. In many cases, EEG signatures have been studied in response to complex, indirect modulators of MA systems, with variations attributed to differences in pharmacological profiles [24,25,26,27]. This limits our ability to draw detailed conclusions about the underlying neurotransmitter mechanisms, especially for substances with multifaceted effects.

Analytically, most studies still rely on the Fourier transform to generate EEG frequency spectra. However, Fourier analysis assumes the EEG signal is stationary—an assumption that is invalid for EEG in general and particularly during pharmacological interventions. In contrast, period-amplitude analysis [28] does not require stationarity and can capture subtle changes in amplitude and signal structure within narrower frequency bands than those provided by conventional Fourier-based power spectra [29]. This approach has been successfully applied to studies of sleep deprivation [30] and the effects of psychotropic drugs on EEG [31].

It’s also worth noting that in order to detect subtle EEG effects of different drugs, narrow-band frequency analysis is often more appropriate. Unfortunately, many studies still rely on broad, traditional frequency bands (delta, theta, alpha, beta), averaging across these wide ranges. This averaging can obscure dynamic EEG changes as they shift over time and cross band boundaries. To overcome this, we developed a statistical clustering method that identifies significant EEG changes in finely detailed time-frequency domains. This method was successfully applied in our previous research [32]; it has been shown to be effective in analysing beta-band EEG activity associated with cognitive function [33].

In this study, we focused on how neurotransmitter-receptor interactions in the MA systems shape the brain’s EEG frequency profile. Using a refined period-amplitude algorithm with high frequency resolution, we examined EEG changes in freely moving rats following intracerebroventricular (i.c.v.) injections of MA receptor agonists and antagonists—administered both separately and in combination, and at varying doses. The i.c.v. route was chosen to bypass the blood-brain barrier (BBB) and ensure direct delivery of the substances into the brain, avoiding their transformation in the bloodstream, which is characteristic of systemic applications. To analyze the EEG data more precisely, we applied our clustering-based statistical method, allowing us to track significant changes in EEG spectra over time and frequency. Those clusters, which were revealed after i.c.v. injection of an agonist and attenuated or completely disappeared after intracerebral pretreatment with the corresponding antagonist, were considered as the EEG spectral “signature” of the analyzed MA system.

## 2. Materials and Methods

### 2.1. Experimental Animals

Twenty-two male Wistar rats from colonies at the University of Glasgow (Scotland, UK) and bred under controlled barrier conditions at the Pushchino Department of the Institute of Bioorganic Chemistry (Pushchino, Russia) were used in this study. The rats of this strain were chosen because of their great popularity in various types of research, from behaviour models to pathway signalling. Mice were housed with a 12-h/12-h light/dark cycle, 22–25° C RT, 50–55% relative humidity, with food and water ad libitum. Experiments were carried out between 8 a.m. and 5 p.m. All manipulations were performed in accordance with the principles enunciated in the “Guide for Care and Use of Laboratory Animals, NIH publication No 85-23”, and with the “Guidelines for accommodation and care of animals and the principles of the Directive 2010/63/EU” on the protection of animals used for scientific purposes. The main effort was made to minimize animal suffering and reduce the number of subjects used.

### 2.2. Electrodes and Cannula Implantation

Adult (8–9-week-old) rats of 290–330 g weight were implanted with cerebral electrodes and cannulas under Nembutal (Pentobarbital, Merck KGaA, Darmstadt, Germany) subcutaneous anaesthesia at an initial dose of 60 mg/kg and its subsequent fractional increase depending on the complexity of the operation and animal condition. After falling asleep (usually in 25–30 min), each rat was softly fixed on the operating table, and the dorsal surface of the head was infiltrated with a local anaesthetic. Two to three minutes later, the skin flap, periosteum, and a part of the muscles on the lateral surface of the skull, 4–5 mm below the cristae, were removed. As necessary, electrocoagulation of damaged blood capillaries was performed, followed by a treatment with hydrogen peroxide on the exposed surface of the skull and its drying. Then each animal was placed in a stereotaxic apparatus modified for our aims, where its skull was oriented and fixed in it in accordance with the requirements of the rat brain atlas used [34]. The coordinates of the points for drilling of the skull surface were chosen and the holes with a diameter of 0.4 mm for the electrodes and 1.2 mm for the intracerebral cannula were prepared.

The electrodes (0.4 mm stainless steel wires) were inserted epidural over the right frontal cortex for EEG recordings (AP −1.5, L 2 mm) [35], whereas reference electrodes were placed close to the midline in the nasal bone. The cannula (stainless needle with 1-mm outer diameter and 11-mm length) was placed into the right lateral ventricle (AP −0.4, L 3.2, DV 3.7, α 20°, where α indicates the angle between the cannula and midline vertical plane) [34]. The electrodes and cannula were anchored to the skull preliminarily with a small amount of hydroscopic “zinc-phosphate cement” (“Vladmiva”, Moscow, Russia). Then, to avoid weakening of the fixation to the skull, its dry surface was covered with a thin layer of this cement to stop the spreading of the liquor able to elevate at times from the brain via bone sutures. After soldering the electrodes to the corresponding pins of a mini-connector (Sullins Connector Solutions, San Marcos, CA, USA), the construction was stabilized on the skull with cement.

During cannula implantation into the lateral ventricles of the brain, its correct positioning was operatively evaluated by the presence of a stable or pulsating drop of the liquor on the cannula tip. At the end of the experiments, angiotensin II (at a dose of 10 μg per rat) was injected through the cannula to assess the adequacy of its tip position in the cerebral ventricle by increased water consumption by the animal [36]. In preliminary experiments, to test the cannula tip location in the ventricle directly, the injection of methylene blue solution at a dose of 6–8 μL per animal was used [37]. Finally, the electrode and cannula positions in the brain were morphologically verified on its slices prepared by use of a freezing microtome (Reichert, Austria) after Nembutal overdose euthanasia.

### 2.3. EEG Recording and Drug Treatment

Animals were housed individually to prevent damage to the implants and to keep the animals away from stressful situations associated with social behaviour in groups. From the fourth day after surgery and for several consecutive days, the rats were adapted, 1 h/day, both to an experimental box (transparent Perspex, 15 × 17 × 20 cm) placed in an electrically shielded chamber and to handling (connecting/disconnecting the animal to/from the recording cable). In the experiments started a week after the surgery, each single rat was placed into the box at least 30 min before EEG recordings. After 10-min baseline EEG recordings, intracerebroventricular (i.c.v.) injections of saline (as a vehicle control) and, with two-day pauses, an agonist at different doses were performed. The EEG effects of agonists vs. saline were analysed for 60 min. Furthermore, this protocol was used for i.c.v. injections of the corresponding antagonist (a list of the drugs is shown in Table 1). Additional control experiments with saline, performed after the series with agonists and antagonists, demonstrated a lack of cumulative or delayed effects of these substances. This approach (each animal is used as its own control) largely eliminates the problems associated with the necessity of preliminary randomisation of animals and especially their individual anatomical and biochemical specificities that have been shown to be important for the studies using a central administration of pharmacological substances [37].

In combined “antagonist + agonist” experiments, an agonist was injected 20 min after the antagonist, whereas in control experiments, saline was used in both. During the baseline period, animals were in a relaxed posture, with closed eyes, and EEG registration was temporarily interrupted when they woke up. This ensured that, before commencing the main part of the experiment, the initial state of each animal was similar to enable correct comparison of the results obtained in different experimental groups. During the injection, 5 μL of a solution was slowly infused (for about 2 min) into the lateral ventricles of the freely moving animal through the guide cannula by use of a 10-μL Hamilton syringe with attached flexible tubing and blunt stainless steel needle (0.8 mm inward diameter). The infusion system was previously sterilized with ethanol and washed with sterile saline; the agonist solutions were freshly prepared by use of a Millipore sterilising filter with a pore diameter of 0.22 μm. After the infusion, the needle inside the guide cannula was replaced by a sterile, stainless steel wire, and the cannula was sealed with a sterile plastic lid. Taking into account a rapid spread of the substance injected into the lateral ventricle throughout the brain [38], EEG recording started immediately after the injection. Experiment duration did not exceed 2–2.5 h. No visible differences in their behaviour were observed after injections of agonists and antagonists at the used doses.

### 2.4. Computation of EEG Spectra

The frequency spectra of successive 12-sec EEG epochs were analysed online in the range of 0.25–30.5 Hz (amplified and band-pass filtered at 0.1–50 Hz) via a computerized system using a modified version of period-amplitude analysis [39]. Each epoch was digitized with a multichannel A/D DT2814 converter (Data Translation, Inc., Marlboro, MA, USA) using a sampling rate of 330 Hz. This program allows selecting any frequency range for analysis, specified by its lower and upper values. In this case, the analogue-to-digital converter automatically detects the signal sampling frequency and determines the maximum possible number of frequency subranges. The integrated amplitudes in twenty selected narrow EEG frequency bands with central frequencies (in Hz) of 0.5, 1.0, 1.5, 2.0, 2.5, 3.0, 3.6, 4.3, 4.9, 5.7, 6.4, 7.2, 8.2, 9.3, 10.4, 11.9, 13.8, 16.4, 20.3, 26.5 and amplitude ratios for each band over the integrated amplitudes in the 0.25–30.5 Hz range were calculated. The bands are marked below by their centre (mean) frequencies (see in brackets). In this case, the sub-range boundaries correspond approximately to the middle between the central frequencies, but without intersection, thus excluding mutual influences of the sub-ranges and ensuring uniformity of the amplitude-frequency characteristic. In addition, as a high quality of the EEG recordings was the main criterion used for including and excluding animals in this study, the program allowed both automatic and manual rejection of EEG fragments containing artefacts. Nevertheless, it should be noted that they were very rarely due to tight connections in the recording cable sockets, eliminating “contact” artefacts and insertion of the cable into a thin and flexible grounded shield protecting the EEG signal from so-called “capacitance” artefacts. Thus, no one animal was excluded from the experiments. (For other details, see [40]).

### 2.5. Statistics

**Figure 1:**
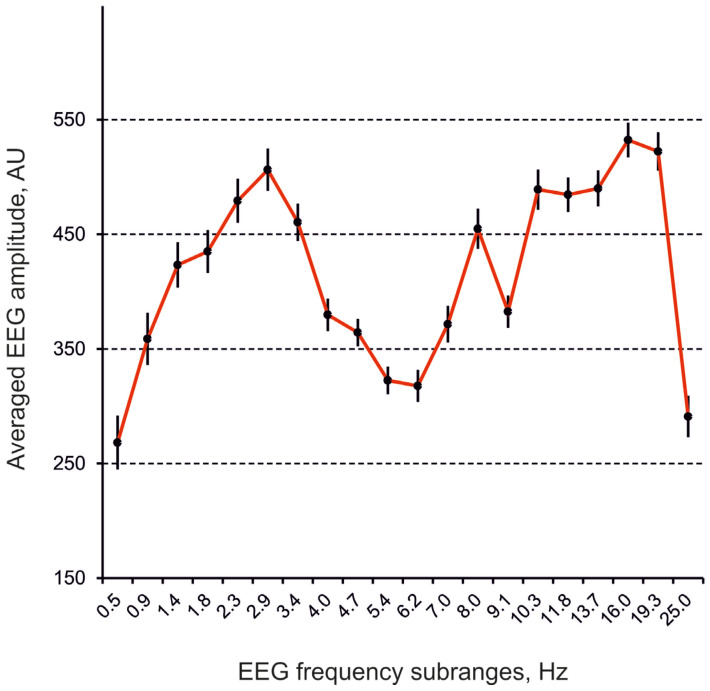
Averaged frequency spectra of EEG from the frontal cortex in 10-min baseline intervals in rats (n = 5). Ordinate is an averaged EEG amplitude in individual frequency sub-range normalized to the sum of the amplitudes in the whole spectrum, in arbitrary units (AU); abscissa indicates different frequency subranges denoted by their central frequencies, in hertz (Hz); vertical bars are ±SEM.

The frequency spectra of individual baseline EEGs (before i.c.v. injections) for 12-s epochs were averaged in 10-min intervals by calculating average values of the amplitudes in each frequency subrange, confidence intervals, and standard errors of the mean for all rats used in the corresponding series (Figure 1). Then the values obtained after i.c.v. injections of a vehicle (saline) or a substance were normalized, taking into account possible differences in baseline EEG spectra recorded in both series. The average values of EEG spectra in corresponding experiments with a substance and a vehicle were compared as a relative difference between them, calculated as [(substance − saline)/saline] × 100% and assessed for reliability by the Mann–Whitney non-parametric U-test at a significant level of *p* < 0.05. To analyse and compare significant effects in the obtained time-frequency matrices, the image processing algorithms were applied. For this purpose, the distributions of calculated values were resized by bilinear interpolation and smoothed out by using a two-dimensional Fourier filtration, followed by selection of areas (“clusters”) separately for positive and negative significant values. Finally, equipotential edge lines were built for these values in each sub-band, which allowed the highlighting of clusters and the estimation of their size (the number of significant differences and the sum of their values) and time-frequency position. For comparison of the extent of significant effects involved in building “clusters” in different experimental conditions, the Mann–Whitney non-parametric U-test at *p* < 0.05 was used. Applied in this study, a dose-effect approach allowed the revealing of both a general and unique influence of the substances on the EEG spectral composition and had the potential for further analysis of unapproved assumptions. For U-test analyses, STATISTICA 10 (StatSoft, Inc., Tulsa, OK, USA) was used, whereas power and effect size were calculated by use of G*Power 3.1.9.4 (http://www.psycho.uniduesseldorf.de/abteilungen/aap/gpower3, assessed on 6 February 2019). At the effect size of 0.75–0.9 and power of 0.8, G*Power showed that the sample sizes chosen for the groups of rats (n = 6 and 8) were reasonable for our EEG study (see below).

## 3. Results

Baseline cortical EEGs recorded in non-narcotized freely moving rats were characterized by dominant delta and alpha-beta activities with peaks of about 2.9 Hz and 16 Hz, respectively (Figure 1).

Pharmacological activation of the monoaminergic systems by the agonists specifically affected EEG composition, depending on the system’s type, which was evidently associated with agonist-receptor interaction. The effects were either attenuated or completely diminished by pretreatment with corresponding antagonists (see below).

### 3.1. Serotoninergic Transmission

**Figure 2:**
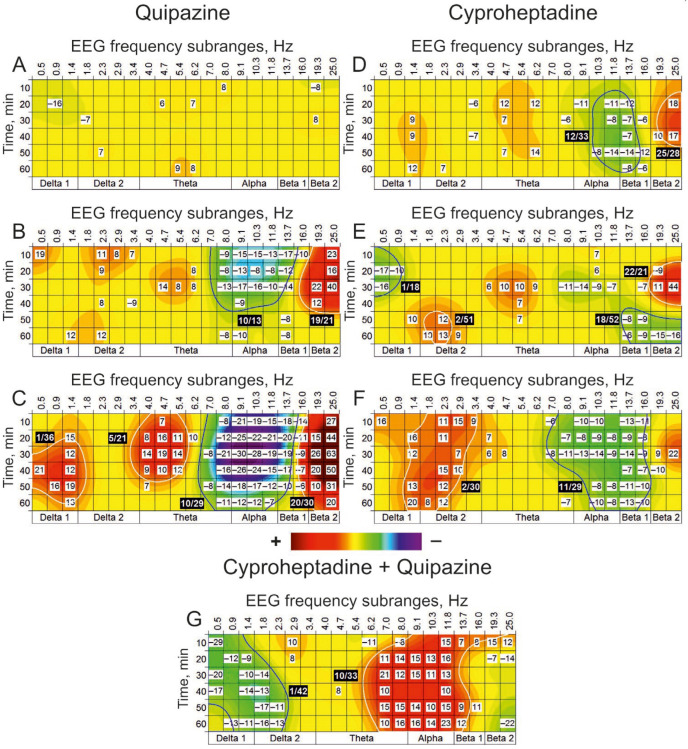
EEG effects of i.c.v. injected quipazine (vs. saline) at doses of 1, 10, and 100 nmol (**A**–**C**) (n = 6) and cyproheptadine at doses of 10, 100, and 1000 nmol (**D**–**F**) (n = 6). On plate G, relative changes in the effects of quipazine at a dose of 100 nmol produced by i.c.v. pretreatment with cyproheptadine at a dose of 10 nmol vs. those with saline pretreatment (n = 6). Cyproheptadine inverted both the (–) and (+) clusters with central frequencies of 1 and 10 Hz (**G**) characteristic for quipazine (**C**). (The main statistical data are presented in Table A1).

Quipazine, an agonist for 5-HT receptors, was ineffective at dose of 1 nmol, initiated the clusters with significantly suppressed and enhanced EEG activities (“(−)” and “(+)” clusters) around 10 Hz and 19 Hz, respectively, at higher dose of 10 nmol, and produced more powerfully expressed effects at maximally used dose of 100 nmol (Figure 2A, B and C, respectively).

Dose-dependent differences (100 nmol vs. 10 nmol) in EEG effects of quipazine were expressed in increased number of the (+) clusters with central frequencies of 1 Hz and 5 Hz and significant enlargement of the (−) and (+) clusters with central frequencies of 10 Hz and 20 Hz, respectively (c.f., Figure 2B,C). At a minimal dose of 10 nmol, cyproheptadine, a 5-HT antagonist, predominantly produced moderate suppression of EEG activity around 12 Hz (Figure 2D). At a maximal dose of 1000 nmol, cyproheptadine significantly increased (approximately three times) the cluster’s size relative to that observed at a minimal dose of 10 nmol (c.f., Figure 2D,F). In addition, a new cluster with enhanced EEG activity around 2 Hz was denoted. Preliminarily i.c.v. injected cyproheptadine at a dose of 10 nmol (Figure 2G) inverted both the (−) and (+) clusters with central frequencies of 1 and 10 Hz, respectively, which were characteristic of quipazine applied alone at the dose of 100 nmol (see Figure 2C). Thus, the EEG oscillations with frequencies characteristic of these clusters seem to be formed with an involvement of 5-HT synaptic mechanisms.

### 3.2. Dopaminergic Transmission

**Figure 3:**
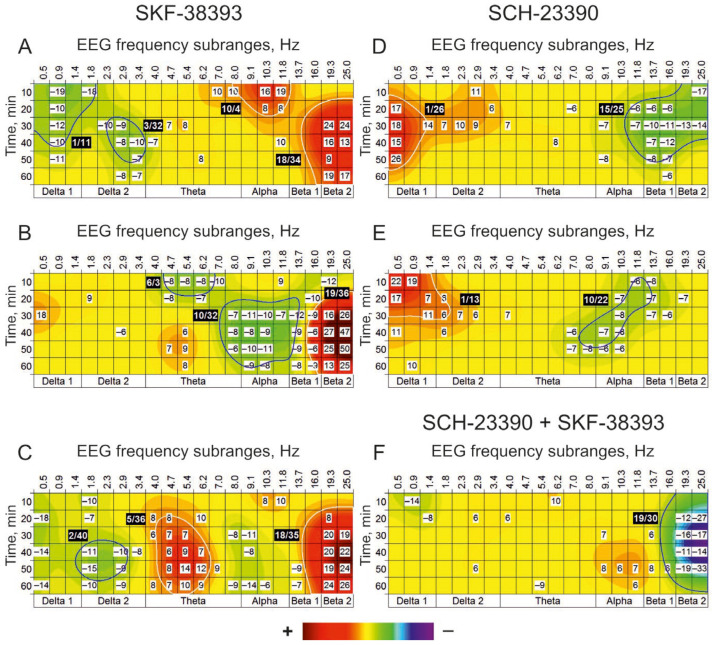
EEG effects of i.c.v. injected SKF-38393 (vs. saline) at doses of 10, 50, and 100 nmol (**A**–**C**) (n = 8) and SCH-23390 at doses of 10 and 100 nmol (**D**,**E**) (n = 8). On plate F, relative changes in the effects of SKF-38393 at a dose of 100 nmol produced by i.c.v. pretreatment with SCH-23390 at a dose of 10 nmol vs. those with saline pretreatment (n = 8). SCH-23390 significantly suppressed the (+) cluster with a central frequency of 18 Hz (**F**), characteristic of SKF-38393 (**C**). (The main statistical data are presented in Table A2).

Centrally (i.c.v.) injected SKF-38393, an agonist for DA receptors, was effective at all used doses of 10, 50, and 100 nmol, producing significant amplification of cortical EEG activity predominantly around 18 Hz (Figure 3A–C). The main differences in the agonist dose-effects were expressed in early (+) cluster with central frequency of 10 Hz at dose of 10 nmol (Figure 2A), delayed the (−) cluster around this frequency at dose of 50 nmol (Figure 2B) and the enhanced EEG activity in vicinity of 5 Hz at maximal dose of 100 nmol (Figure 2C). An antagonist for DA receptors, SCH-23390, produced practically similar changes in EEG spectra at both doses of 10 and 100 nmol, forming the (+) cluster around 1 Hz and the (−) clusters with central frequencies of 15 and 10 Hz, respectively (Figure 3D,E). In combined “antagonist + agonist” experiments, preliminarily, i.c.v. Injected SCH-23390 at a dose of 10 nmol (Figure 3F) significantly suppressed the (+) cluster with a central frequency of 18 Hz, characteristic of SKF-38393 applied alone at a dose of 100 nmol (see Figure 3C). Thus, the EEG oscillations with frequencies characteristic of these clusters seem to be formed with an involvement of DA synaptic mechanisms.

### 3.3. Noradrenergic Transmission

**Figure 4:**
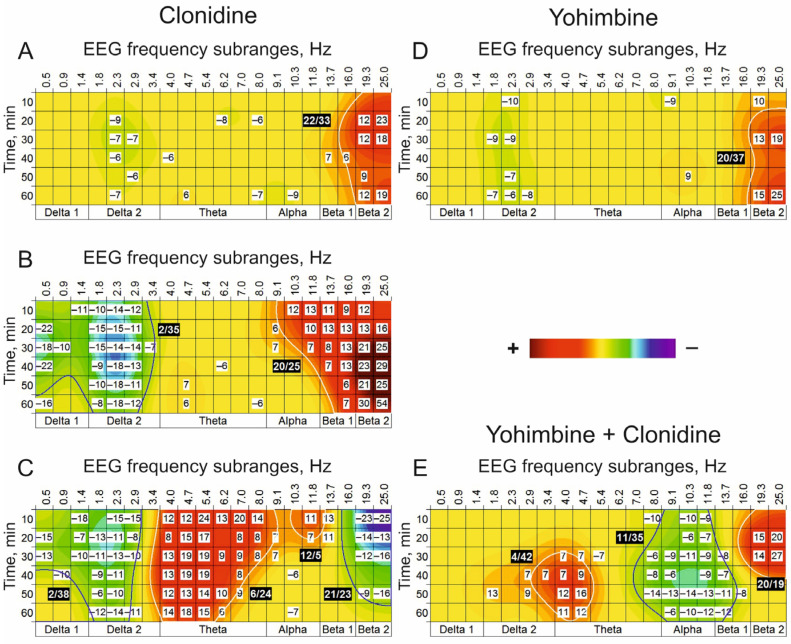
EEG effects of i.c.v. injected clonidine (vs. saline) at doses of 1, 10, and 100 nmol (**A**–**C**) (n = 8) and yohimbine at a dose of 10 nmol (**D**) (n = 8). On plate E, relative changes in the effects of clonidine at the dose of 100 nmol produced by i.c.v. pretreatment with yohimbine at a dose of 10 nmol vs. those with saline pretreatment (n = 8). Yohimbine amplified the (+) cluster around 6 Hz and diminished the (−) cluster in the vicinity of 21 Hz (**E**), which were induced by clonidine (**C**). (The main statistical data are presented in Table A3.)

Centrally (i.c.v.) injected clonidine, an agonist for NE receptors, formed the (+) cluster with central frequency around 22 Hz at a minimally used dose of 1 nmol (Figure 4A) that was significantly enlarged and accompanied by the (–) cluster around 2 Hz at a dose of 10 nmol (Figure 4B).

Maximally used dose of 100 nmol was supportive for this (−) cluster and initiated an additional (+) cluster with a central frequency of 6 Hz and one more (−) cluster around 21 Hz (Figure 4C). Interestingly, an antagonist for NE receptors, yohimbine, at a dose of 10 nmol, produced the (+) cluster similar to that at the minimal dose of the agonist (c.f., Figure 4A,D). Nevertheless, the antagonist pretreatment significantly diminished the (+) cluster around 6 Hz and the (−) cluster in the vicinity of 21 Hz produced by clonidine at a dose of 100 nmol (c.f., Figure 4C,E). These allow the suggestion that the EEG oscillations with frequencies characteristic of these clusters produced by clonidine seem to be formed with an involvement of NE synaptic mechanisms.

## 4. Discussion

In cortical EEG spectra, dose-dependent effects of agonists and antagonists for receptors of serotonin (5-HT), dopamine (DA), and norepinephrine (NE), centrally (i.c.v.) applied alone and in the “antagonist+agonist” composition, were studied by use of the “time-frequency” clustering. With this approach, several specific clusters were revealed after injections of different agonists, which in turn were either attenuated or eliminated by intracerebral pretreatment with corresponding antagonists. These peculiar areas in the EEG spectra were considered as corresponding “signatures” of the analyzed MA systems.

*Serotoninergic transmission*

Indeed, quipazine, an agonist for 5-HT_1,2_ receptors, has been shown to form the (−)cluster of suppressed EEG activity centred around 10 Hz in the alpha band with flanks in the theta and beta_1_ bands (Figure 2B,C). This agonist’s effect was inverted, making the (+) cluster after the pretreatment with an antagonist for 5-HT_1,2_ receptors, cyproheptadine (Figure 2G), which highlights a specific involvement of the 5-HT system in the modulation of EEG activity in this frequency range. Furthermore, activated by quipazine 5-HT_1_ subtype of serotonin receptors seems to be responsible for this suppressive EEG effect because of their inhibitory role for neuronal activity [41]. On the other hand, 5-HT_2_ receptors are supposedly involved in the modulating effects of quipazine in the delta band, given evident clusters centred around 1 Hz of both suppressed EEG activity after pretreatment with cyproheptadine (Figure 2G) and enhanced EEG activity after injection of agonist at a maximal dose of 1000 nmol (Figure 2C). Together with the main (−) cluster centred around 10 Hz, these specify a direct involvement of 5-HT receptors in the formation of the EEG frequency subranges that is in line with the results of electrophysiological analysis of the functioning of different subtypes of 5-HT receptors [42]. Interestingly, the (+) cluster produced by quipazine in the vicinity of 20 Hz (Figure 2B,C) was insensitive to cyproheptadine (Figure 2G). This non-specific effect was observed in experiments with systemic injections of 5-HT-mimetics as well [10], which may explain some contradictory results in the field [43] and attract attention to the studies of sleep stages [44] and regulation of epileptic activity [45]. Together, the data obtained in this study pointed out the involvement of 5-HT receptors in the formation of EEG frequency composition and the necessity of taking into account the role of different subtypes of 5-HT receptors in the effects of substances affecting their activity.

*Dopaminergic transmission*

An agonist for DA receptors, SKF-38393, has been shown to form a cluster of enhanced EEG activity (the (+) cluster) around 18 Hz at all used doses (Figure 3A–C) that was inverted by the antagonist, SCH-23390, pretreatment (Figure 3F). These directly support a suggestion about the involvement of DA-modulated networks in the generation of cortical beta oscillations [46] and, in turn, their role in cognition [33]. Given this, the “beta” (+) cluster, induced by quipazine but insensitive to cyproheptadine (see above), may be considered as a consequence of a “serotonin-dopamine” interaction widely spreading in the brain [47]. This, together with the cyproheptadine-sensitive “alpha” cluster (Figure 2C), highlights a role of 5-HT receptors in the formation of the beta (through the DA receptors) and alpha (through the 5-HT receptors) activities involved in memory mechanisms [48]. These results are in line with those obtained at systemic application of SKF 38393 [49] and during stimulation of endogenous DA release by amphetamine [50].

*Noradrenergic transmission*

More complicated and mixed results presented in this section need more detailed analysis. Firstly, the dose-dependent changes in EEG beta activity are non-linear: the (+) clusters at lower doses of clonidine (Figure 4A,B) were replaced by the beta_1_ (−) cluster at the highest dose of 100 nmol (Figure 4C). The latter effect was similar to that observed in experiments with the injection of clonidine in the third cerebral ventricle [51]. Secondly, a central (theta) area insensitive to lower doses was tremendously affected by the highest dose of the agonist (Figure 4C, the (+) cluster). Thirdly, at enhanced doses (10 and 100 nmol) of clonidine, the delta (−) clusters were developed (Figure 4B,C). Fourthly, yohimbine at a dose of 10 nmol (Figure 4D) produced the beta (+) cluster about similar to that after i.c.v. injection of clonidine at a minimal dose of 1 nmol (Figure 4A) that is in line with results of analogous earlier experiments [8]. Finally, a very interesting and complicated phenomenon is a local amplification of the EEG agonist effects expressed as the (+) clusters around 4 Hz and 20 Hz after the antagonist pretreatment (Figure 4E). This may be associated with a composition of several subclasses of alpha_2_ NE receptors [52] and their presynaptic types [53]. Indeed, yohimbine is well known to antagonize α2-autoreceptors in the locus coeruleus, thus stimulating NE release by hyperpolarizing presynaptic rather than postsynaptic alpha_2_ receptors (see, e.g., [54]. That explains the initiation of the beta_2_ (+) cluster by yohimbine applied alone (Figure 4D) and local amplification of the theta (+) cluster and inversion of the clonidine effect in the beta_2_ band (Figure 4E). Together, these specify a direct involvement of NE receptors in the formation of these EEG frequency subranges.

Thus, the specific involvement of 5-HT, DA, and NE receptors in the generation of definite EEG oscillations and their possible interplay supposedly is associated with cerebral mechanisms supporting efficacy and selectivity of neuronal communications in the normal brain [46] and at its pathology [27,55]. Thus, a fine neuronal and network organisation of the interaction between MA systems needs to be investigated in further studies in this area [56]. Furthermore, EEG data obtained in the chosen format for other neurotransmitter systems (GABAergic, glutamatergic, and cholinergic) and interrelations between them in a row of common disorders [23,57,58,59] should be analysed for a detailed understanding of those adaptive mechanisms that might be involved in their treatment.

## 5. Conclusions

In this study, centrally (i.c.v.) injected agonists of the monoaminergic systems (serotoninergic, dopaminergic, and noradrenergic) produced clusters of significant changes specifically distributed in the cortical EEG frequency spectra. The association of the effects with direct activation of the receptors was supported by their attenuation or elimination by i.c.v. pretreatment with appropriate antagonists. The links between the cluster features in EEG frequency spectra and activities of different monoaminergic systems may be extremely useful for an analysis of neurotransmitter mechanisms of treating the diseased brain with substances penetrating the blood-brain barrier, thus, potentially applicable for patients in clinics. The approach developed in this study allows (a) the revealing of pathological changes in the functioning of cerebral neurotransmitter systems, (b) the determination of further pharmacological treatment, and (c) the monitoring of the efficacy of chosen therapeutic tactics. The obtained results may be useful in the analysis of (a) brain disorders associated with malfunctioning of monoaminergic systems, (b) neurochemical mechanisms of new/unfamiliar substances, and (c) the involvement of the neurotransmitter systems in effects of environmental disturbances associated, in particular, with fluctuations in characteristics of magnetic fields and microwave irradiation.

## Figures and Tables

**Table 1:** The substances used in this study.

Substances-Analyzers Used in this Study for MA Receptors		
**Full Name**	**Short Name**	Affinity
Serotonin (5-HT)		
Quipazine dimaleate	Quipazine	Agonist (5-HT_1,2_)
Cyproheptadine hydrochloride	Cyproheptadine	Antagonist (5-HT_1,2_)
Dopamine (DA)		
(±)-SKF-38393 hydrochloride	SKF-38393	Agonist (DA_1_)
(+)-SCH-23390 hydrochloride	SCH-23390	Antagonist (DA_1_)
Norepinephrine (NE)		
Clonidine hydrochloride	Clonidine	Agonist (α_2_)
Yohimbine hydrochloride	Yohimbine	Antagonist (α_2_)

Sources: quipazine, (±)-SKF-38393 hydrochloride, clonidine, and (+)-SCH-23390 hydrochloride are from Sigma-Aldrich (St. Louis, MO, USA); cyproheptadine-from Serva (Denver, CO, USA); yohimbine-from Reckitt & Colman (Slough, UK).

## Data Availability

Raw data supporting the results is available on request from the first author (V.V.).

## Appendix A

**Table A1:** Time-frequency distribution of relative differences in cortical EEG spectra averaged in 10-min intervals (indicated in the first column) in six rats after i.c.v. injections of Quipazine (A) and its modification by preliminarily injected Cyproheptadine (B). EEG “classical” frequency bands are denoted in the first horizontal line. In the second one, the mean values of individual frequency sub-ranges are presented. Colored cells indicate significantly (*p* < 0.05, U-test) elevated (red) and lowered (blue) values.

Quipazine (100 nmol) vs. Saline																				
A	Delta 1			Delta 2				Theta						Alpha			Beta 1		Beta 2	
Hz Min	0.5	0.9	1.4	1.8	2.3	2.9	3.4	4.0	4.7	5.4	6.2	7.0	8.0	9.1	10.3	11.8	13.7	16.0	19.3	25.0
10	16	10	6	0	1	4	2	4	7	3	0	0	−8	−21	−19	−15	−18	−14	4	27
20	6	−2	15	0	1	−2	0	8	16	11	10	1	−12	−25	−22	−21	−20	−11	15	44
30	5	2	12	0	−3	1	2	14	19	14	1	−8	−21	−30	−28	−19	−19	−9	26	63
40	21	1	12	−1	0	−1	−1	9	10	12	0	−5	−16	−26	−24	−15	−17	−7	20	50
50	7	16	19	1	0	1	0	7	4	4	−1	−8	−14	−18	−17	−12	−10	−6	10	31
60	13	2	13	0	0	−2	−1	1	1	2	−1	−3	−11	−12	−12	−7	0	1	4	20
Cyproheptadine (10 nmol)+ Quipazine (100 nmol) vs. Saline + Saline																				
B	Delta 1			Delta 2				Theta						Alpha			Beta 1		Beta 2	
Hz Min	0.5	0.9	1.4	1.8	2.3	2.9	3.4	4.0	4.7	5.4	6.2	7.0	8.0	9.1	10.3	11.8	13.7	16.0	19.3	25.0
10	−29	−1	0	−6	0	10	2	1	−3	−3	−11	−6	−8	−1	0	15	7	8	15	12
20	−13	−12	−9	−4	2	8	1	0	−1	−2	0	11	14	15	13	16	5	0	−7	−14
30	−20	−6	−10	−14	0	0	0	1	2	0	3	21	12	15	11	13	0	0	−2	−13
40	−17	−1	−14	−13	−2	0	2	1	8	0	1	10	2	0	0	10	0	0	8	2
50	−9	−6	−7	−17	−11	0	0	−1	0	−3	0	15	15	14	10	15	9	11	1	−11
60	−1	−13	−11	−16	−13	0	1	0	0	0	0	10	16	16	14	23	12	3	−1	−22

**Table A2:** Time-frequency distribution of relative differences in cortical EEG spectra averaged in 10-min intervals (indicated in the first column) in six rats after i.c.v. injections of SKF-38393 (A) and its modification by preliminarily injected SCH-23390 (B). Colored cells indicate significantly (*p* < 0.05, U-test) elevated (red) and lowered (blue) values.

SKF-38393 (100 nmol) vs. Saline																				
A	Delta 1			Delta 2				Theta						Alpha			Beta 1		Beta 2	
Hz Min	0.5	0.9	1.4	1.8	2.3	2.9	3.4	4.0	4.7	5.4	6.2	7.0	8.0	9.1	10.3	11.8	13.7	16.0	19.3	25.0
10	−9	−3	−6	−10	0	0	0	4	0	0	4	2	2	1	8	10	0	0	0	−3
20	−18	−5	0	−7	0	−2	−1	8	8	5	10	3	−2	−3	0	−1	−4	−2	8	7
30	−7	−3	0	−4	−3	−4	0	6	7	7	0	−1	−8	−11	−1	−1	−3	0	20	19
40	−14	−3	1	−11	−4	−10	−8	5	6	9	7	3	−2	−8	−1	0	−2	0	20	22
50	−8	−3	−1	−15	−4	−9	−5	3	8	14	12	9	0	−4	−3	−4	−9	−5	19	24
60	−14	−1	0	−10	−3	−9	−2	8	7	10	9	0	−9	−14	−6	−1	−7	0	24	26
SCH-23390 (10 nmol)+ SKF-38393 (100 nmol) vs. Saline + Saline																				
B	Delta 1			Delta 2				Theta						Alpha			Beta 1		Beta 2	
Hz Min	0.5	0.9	1.4	1.8	2.3	2.9	3.4	4.0	4.7	5.4	6.2	7.0	8.0	9.1	10.3	11.8	13.7	16.0	19.3	25.0
10	0	−14	−4	−3	−1	0	1	4	0	0	10	7	1	1	0	2	0	1	0	−1
20	−1	−4	−8	−3	0	6	3	6	1	0	0	3	5	3	2	3	4	2	−12	−27
30	−1	−3	−5	0	1	1	1	1	0	−1	3	1	3	7	2	5	6	3	−16	−17
40	−1	6	−4	−2	5	4	1	1	0	−2	0	0	2	1	2	2	1	0	−11	−14
50	0	−3	0	0	3	6	3	0	−5	−5	−1	0	4	8	6	7	8	6	−19	−33
60	1	−1	−2	0	2	3	1	2	0	−9	0	−1	0	2	0	6	4	4	−6	−7

**Table A3:** Time-frequency distribution of relative differences in cortical EEG spectra averaged in 10-min intervals (indicated in the first column) in six rats after i.c.v. injections of Clonidine (A) and its modification by preliminarily injected Yohimbine (B). Colored cells indicate significantly (*p* < 0.05, U-test) elevated (red) and lowered (blue) values.

Clonidine (100 nmol) vs. Saline																				
A	Delta 1			Delta 2				Theta						Alpha			Beta 1		Beta 2	
Hz Min	0.5	0.9	1.4	1.8	2.3	2.9	3.4	4.0	4.7	5.4	6.2	7.0	8.0	9.1	10.3	11.8	13.7	16.0	19.3	25.0
10	0	−6	−18	−6	−15	−15	−6	12	12	24	13	20	14	6	5	11	13	0	−23	−25
20	−15	−2	−7	−13	−11	−8	1	8	15	17	4	8	8	7	0	7	11	0	−14	−13
30	−13	−3	−10	−11	−13	−10	0	13	19	19	9	9	8	7	0	3	3	0	−12	−16
40	−6	−10	0	−9	−11	−5	0	13	19	19	0	8	1	0	−6	0	1	2	−4	−1
50	0	−2	−2	−6	−10	−5	0	12	13	14	10	9	2	0	−1	0	2	−1	−9	−16
60	0	0	−3	−12	−14	−11	0	14	18	15	6	5	0	−1	−7	0	1	1	0	0
Yohimbine (10 nmol)+ Clonidine (100 nmol) vs. Saline + Saline																				
B	Delta 1			Delta 2				Theta						Alpha			Beta 1		Beta 2	
Hz Min	0.5	0.9	1.4	1.8	2.3	2.9	3.4	4.0	4.7	5.4	6.2	7.0	8.0	9.1	10.3	11.8	13.7	16.0	19.3	25.0
10	9	1	0	6	0	0	1	1	0	−2	3	0	−10	−2	−10	−9	−3	−5	2	8
20	3	0	−3	2	−1	0	−1	1	6	−4	3	0	−3	−5	−6	−7	−4	1	15	20
30	−1	1	0	4	2	0	0	7	7	−7	0	0	−6	−9	−11	−9	−8	0	14	27
40	6	0	0	1	−4	7	7	7	9	−1	2	−2	−8	−6	−3	−9	−7	0	2	0
50	2	−1	5	13	3	9	5	12	16	1	1	0	−14	−13	−14	−13	−11	−8	1	3
60	1	9	8	1	−4	1	4	11	12	2	2	−1	−4	−6	−10	−12	−12	0	0	2
